# Putting bicarbonate on the spot: pharmacological insights for CFTR correction in the airway epithelium

**DOI:** 10.3389/fphar.2023.1293578

**Published:** 2023-12-11

**Authors:** Miroslaw Zajac, Agathe Lepissier, Elise Dréano, Benoit Chevalier, Aurélie Hatton, Mairead Kelly-Aubert, Daniela Guidone, Gabrielle Planelles, Aleksander Edelman, Emmanuelle Girodon, Alexandre Hinzpeter, Gilles Crambert, Iwona Pranke, Luis. J. V. Galietta, Isabelle Sermet-Gaudelus

**Affiliations:** ^1^ INSERM U1151, Institut Necker Enfants Malades, Paris, France; ^2^ Université de Paris-Cité, Paris, France; ^3^ Centre de Référence Maladie Rare Pour La Mucoviscidose et Maladies de CFTR, Hôpital Necker Enfants Malades, Assistance Publique Hôpitaux de Paris, Paris, France; ^4^ Department of Physics and Biophysics, Institute of Biology, Warsaw University of Life Sciences, Warsaw, Poland; ^5^ Telethon Institute of Genetics and Medicine, Pozzuoli, Italy; ^6^ U1138/CNRS ERL 8228, Centre de Recherche des Cordeliers, Paris, France; ^7^ Service de Médecine Génomique des Maladies de Système et d’Organe, Hôpital Cochin, Paris, France; ^8^ European Reference Network for Rare Diseases, Frankfurt, Belgium

**Keywords:** ion transport across membrane, cystic fibrosis transmembrane conductance regulator, bicarbonate, inflammation, TRIKAFTA

## Abstract

**Introduction:** Cystic fibrosis (CF) is caused by defective Cystic Fibrosis Transmembrane Conductance Regulator (CFTR) proteins. CFTR controls chloride (Cl^−^) and bicarbonate (HCO_3_^−^) transport into the Airway Surface Liquid (ASL). We investigated the impact of F508del-CFTR correction on HCO_3_^−^ secretion by studying transepithelial HCO_3_^−^ fluxes.

**Methods:** HCO_3_^−^ secretion was measured by pH-stat technique in primary human respiratory epithelial cells from healthy subjects (WT) and people with CF (pwCF) carrying at least one F508del variant. Its changes after CFTR modulation by the triple combination VX445/661/770 and in the context of TNF-α+IL-17 induced inflammation were correlated to ASL pH and transcriptional levels of CFTR and other HCO_3_^−^ transporters of airway epithelia such as SLC26A4 (Pendrin), SLC26A9 and NBCe1.

**Results:** CFTR-mediated HCO_3_^−^ secretion was not detected in F508del primary human respiratory epithelial cells. It was rescued up to ∼ 80% of the WT level by VX-445/661/770. In contrast, TNF-α+IL-17 normalized transepithelial HCO_3_^−^ transport and increased ASL pH. This was related to an increase in SLC26A4 and CFTR transcript levels. VX-445/661/770 induced an increase in pH only in the context of inflammation. Effects on HCO_3_^−^ transport were not different between F508del homozygous and F508del compound heterozygous CF airway epithelia.

**Conclusion:** Our studies show that correction of F508del-CFTR HCO_3_^−^ is not sufficient to buffer acidic ASL and inflammation is a key regulator of HCO_3_^−^ secretion in CF airways. Prediction of the response to CFTR modulators by theratyping should take into account airway inflammation.

## 1 Introduction

The airway epithelium interfaces between the internal milieu and the external environment. It is lined by a thin layer of fluid called the airway surface liquid (ASL), whose composition is finely tuned. There is increasing evidence that defects in ASL pH regulation are associated with various respiratory diseases, including cystic fibrosis (CF) (Coakley et al., 2003). CF is a life-limiting disease caused by mutations in the cystic fibrosis transmembrane conductance regulator (CFTR) gene. The most frequent mutation, p. Phe508del, F508del thereafter, is carried at least on one allele by around 80% of people with CF (pwCF) worldwide and results in defective CFTR protein trafficking due to protein misfolding, reduced stability at the cell surface, and dysfunctional channel gating (Pranke et al., 2019).

CFTR transports two of the most abundant and physiologically important anions, namely, chloride (Cl^−^) and bicarbonate (HCO_3_^−^) into the ASL (Quinton, 2008). HCO_3_^−^ plays a key role in epithelial surface homeostasis by ensuring adequate water content, controlling the volume and pH of the periciliary layer, and regulating mucin biophysical properties such as hydration. All these factors are necessary for efficient mucociliary clearance (MCC) (Quinton, 2008; Bridges, 2012; Gorrieri et al., 2016; Tang et al., 2016; Ferrera et al., 2021). Mutations of the CFTR channel have mainly been characterized by their disruption of Cl^−^ transport; however, they also display defective HCO_3_^−^ transport, which is intimately related to CF disease (Quinton, 2001). At the pulmonary level, deficient HCO_3_^−^ secretion was shown to decrease ASL hydration and reduce its pH, which further compromises mucin formation and mucociliary clearance and impairs the airway host defenses (Garland et al., 2013; Hoegger et al., 2014; Shah et al., 2016; Simonin et al., 2019). The addition of HCO_3_^−^ onto the CF epithelium was shown to increase ASL height and pH (Garland et al., 2013), improve mucus viscoelastic properties (Gomez et al., 2020; Ferrera et al., 2021), restore bacterial killing (Pezzulo et al., 2012), and impede the growth and biofilm formation of pulmonary bacterial pathogens (Dobay et al., 2018).

Highly effective CFTR modulator therapy (HEMT) enables partial F508del-CFTR functional restoration. Triple combination HEMT combines two corrector molecules, namely, elexacaftor (VX-445) and tezacaftor (VX-661), to process misfolded F508del-CFTR protein to the cell membrane and a potentiator, ivacaftor (VX-770), to increase channel opening. Phase 3 clinical trials with elexacaftor/tezacaftor/ivacaftor (ETI) have shown dramatic improvement in lung disease and a strong positive impact on the quality of life for patients carrying at least one F508del-CFTR allele (Middleton et al., 2019). This involves an improvement in CFTR-dependent Cl^−^ transport, resulting in better hydration of airway secretions (Morrison et al., 2022). Interestingly, Rehman et al. showed that ETI induced an increase in pH, but this was observed only in the context of inflammation with the addition of TNF-α+IL-17 to the CF epithelium (Rehman et al., 2020; Rehman et al., 2021). According to the authors, the failure of F508del-CFTR correction to improve pH value in the absence of inflammation was unexpected and suggested that CFTR-dependent HCO_3_^−^ secretion and its role in ASL pH control were dependent on the inflammatory status.

These studies, however, relied on ASL pH variation, which involves both transepithelial HCO_3_^−^ and H^+^ flux. To dissect the role of CFTR on pH homeostasis and the potential impact of CFTR correction in the CF lung environment, we characterized HCO_3_^−^ transepithelial flux and ASL pH changes in healthy and CF epithelium and evaluated the impact of the F508del-CFTR rescue in the context of inflammation.

## 2 Materials and methods

### 2.1 Patients

Respiratory airway cell sampling was performed in CF patients and healthy subjects (WT) (Clinical Trials: NCT02965326). The study was approved by the Ile de France 2 Ethics Committee, and written informed consent was obtained from each adult and both parents for participants below 18 years of age (AFSSAPS (ANSM) B1005423-40, EudraCT no. 2010-A00392-37; CPP IDF2: 2010-05-03-3).

### 2.2 Cell culture

Human bronchial epithelial cells were isolated from bronchial explants (one to two bifurcations) by enzymatic digestion as previously described (Golec et al., 2022). Cells were first grown in T75 cm^2^ flasks in DMEM/F-12 cell culture medium supplemented with 10% newborn calf serum, Rho kinase inhibitor Y-27632 (10 μM), piperacillin/tazobactam 90µg/10 μg/mL, colimycin 16 μg/mL, and amphotericin B 5 μg/mL, and additional growth factors. For air–liquid interface (ALI) culture, 350,000 human respiratory airway expanded cells suspended in an amplification medium were seeded on type IV collagen-coated porous filter with a 0.33 cm^2^ surface (6.5 mm diameter, 0.4 μm pore size, Corning, Tewksbury, MA). UG2% medium (DMEM/F-12, supplemented with 2% Ultroser G) containing antibiotics, as mentioned above, was added to the basal side of the filters. After 72 h of liquid/liquid interface, apical media were removed, and cells were cultured in ALI with UG2% basal medium changed every 48–72 h for 3–4 weeks to establish a differentiated epithelium. The transepithelial electrical resistance (RT) of cultures was measured using a chopstick voltmeter (Millicell ERS).

Prior to functional studies, cells were incubated at the basolateral side for 48 h with DMSO 0.03% (referred to as the control condition) or VX-770 (100 nM), VX-445 (3 µM), and VX-661 (3 µM), referred to as VX-445/661/770 (Selleckchem), all dissolved in DMSO. To assess inflammation-induced responses, epithelia were treated on the basolateral side with 10 ng/mL TNF-α (R&D Systems) and 20 ng/mL IL-17 (R&D Systems) for 48 h.

### 2.3 Measurement of HCO_3_^−^ fluxes

To investigate HCO_3_^−^ transepithelial transport, HCO_3_^−^ secretion rates were monitored in Ussing chambers under open-circuit conditions by the pH-stat method. A mini pH electrode connected to an automated titration workstation, a temperature sensor, and a burette (all from Radiometer Analytical) were introduced to the mucosal chamber to automatically titrate 5 mM HCl to maintain the pH of the solution at 7.000 ± 0.005. TitraMaster 85 software was used to control the rate of titration, amount of titrant added, and continuous measurement of solution pH. The rate of HCO_3_^−^ secretion [µEq∙h-1∙cm^-2^] was calculated in 5-min intervals by noting the amount of titrant added, its concentration, and the cell surface area. HCO_3_^−^ secretion rates were calculated 20–40 min after the addition of each activator/inhibitor and considered stable when remaining constant for at least 15 min. The solution composition used for pH-stat measurements is provided in Supplementary Table S1. The apical solution was vigorously bubbled with pure O_2_, while the basolateral solution was bubbled with carbogen (95% O_2_ and 5% CO_2_). The HCO_3_^−^ secretion rates were monitored in the presence of the ENaC channel blocker amiloride (100µM, Spectrum Chemical) (amiloride secretion rate) and after the subsequent addition of activators or inhibitors of ion-transporting proteins to determine their involvement in HCO_3_^−^ transport across the epithelium. This included cAMP agonists forskolin (Fsk) (10µM, Sigma-Aldrich), M3-isobutyl-1-methylxanthine (IBMX) (100µM, Sigma-Aldrich Merck), and genistein (10µM, Sigma-Aldrich Merck) to activate and potentiate CFTR activity, the specific CFTR inhibitor Inh-172 (10µM, Sigma-Aldrich Merck), and in the case of remaining HCO_3_^−^ flux, the SLC26A4 inhibitor YS-01 (5µM, AOBIOUS) and GlyH-101 (10µM, Sigma-Aldrich Merck). This allowed evaluation of the cAMP-activated HCO_3_^−^ flux (Fsk/IBMX + genistein) and its inhibition by Inh-172, GlyH-101, and YS-01. As CFTR may be partially activated at the basal state, the change after CFTR inhibition was considered the index of CFTR activity.

### 2.4 Measurement of ASL pH

In order to measure ASL pH under physiological conditions, we designed a system with a controlled atmosphere enclosure enabling us to maintain humidity, pCO_2_ at 5%, and temperature at 37°C as previously described (Simonin et al., 2019). ASL pH measurements were performed in the presence of the cAMP agonist Fsk (10µM, Sigma-Aldrich), and IBMX (100µM, Sigma-Aldrich Merck) was added to the basolateral solution to stimulate CFTR. As the ASL layer was too thin to allow direct and reproducible measurement of pH without risking disruption, the pH was measured after the addition of the 50 µL solution at the cell monolayer’s apical side. The solution composition was used for physiological conditions, and Cl^−^-free conditions are provided in Supplementary Table S2. This 50 µL solution, representing diluted ASL, was harvested after 8 h of incubation, and the pH was measured immediately, directly in the controlled atmosphere enclosure with a micro-combination pH electrode (Thermo Fisher Scientific Orion 9810BN, Illkirch, Grand Est, France). Prior to each experiment, the pH microelectrode was calibrated with pH 4 and pH 7 buffers. Experimental solutions were equilibrated at pH 7.4. We previously showed that in this setup, the measured pH value did not differ by more than 0.03 pH units from the theoretical one calculated according to the Henderson–Hasselbalch equation (Simonin et al., 2019).

### 2.5 RT–PCR genetic analysis

A measure of 200 ng of total RNA was reverse-transcribed (Thermo Fischer Scientific). Real-time quantitative PCR was performed on a LightCycler (Roche Diagnostics) using the LightCyclerFastStart DNA Master SYBR Green 1 kit (Roche Diagnostics) according to the manufacturer’s protocols, except that the reaction volume was reduced 2.5-fold. Specific primers were designed using Primer 3 (free online software; Supplementary Table S3). In each run, a standard curve was obtained using a serial dilution of stock cDNA prepared from a mix of different samples of total RNA. The expression of the CFTR, SLC26A4, NBCE1, and SLC26A9 transcripts were normalized to cyclophilin-A (PPIA) expression (the mean threshold cycle for PPIA = 27.5 ± 0.1).

### 2.6 Statistical analysis

Statistical analysis was performed using GraphPad Prism^®^ software. Data are presented as mean ± (SD), and the “n\” value represents one filter. Comparisons were performed with the Wilcoxon matched-pair signed-rank test or Mann–Whitney test as appropriate. Correlations were assessed with Spearman’s test. A significant value was retained if *p* < 0.05.

## 3 Results

### 3.1 VX-445/661/770 increases the CFTR HCO_3_^−^ flux

**FIGURE 1:**
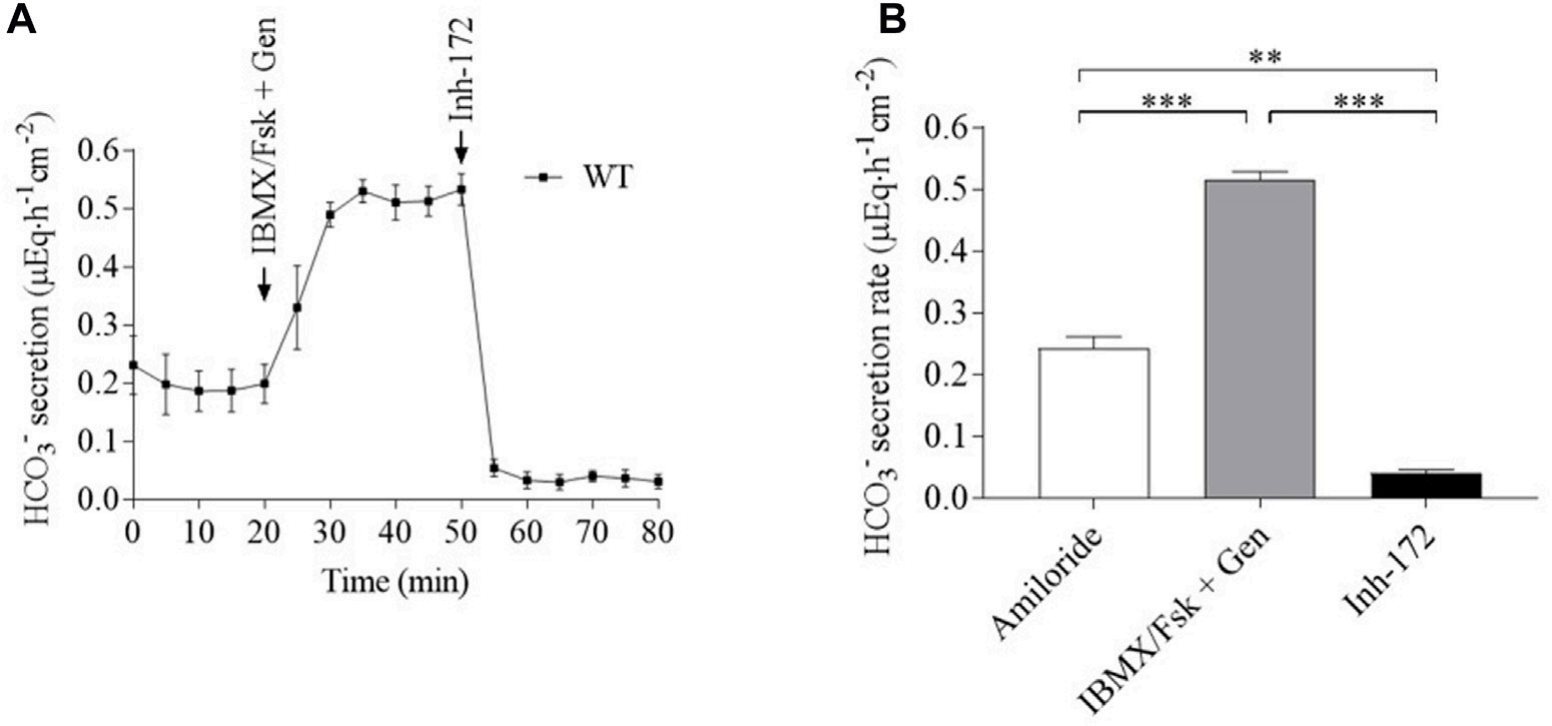
Bicarbonate secretion in the respiratory epithelium expressing WT CFTR. HCO_3_^−^ flux is measured in the WT human respiratory epithelium by pH-stat in the presence of amiloride and after the successive addition of Fsk/IBMX (10µM/100 µM) + genistein (Gen) (100 µM) and Inh-172 (10 µM). **(A)** Representative tracing and **(B)** summary of five experiments. Results are presented as mean ± SD (n = 5). A comparison was carried out by the Wilcoxon matched-pair signed-rank test.**p < 0.01; ***p < 0.001.

pH-stat experiments enabled us to quantify the HCO_3_^−^ secretion rate. Reference HCO_3_^−^ secretion rates in the wild-type (WT) respiratory epithelium are shown in Figure 1 and Supplementary Table S4. Amiloride secretion rate across WT respiratory cells (0.24 ± 0.04 µEq∙h^-1^∙cm^-2^) was increased by Fsk/IBMX + genistein (up to 0.51 ± 0.04µEq∙h^-1^∙cm^-2^) with subsequent complete inhibition by Inh-172.

**FIGURE 2:**
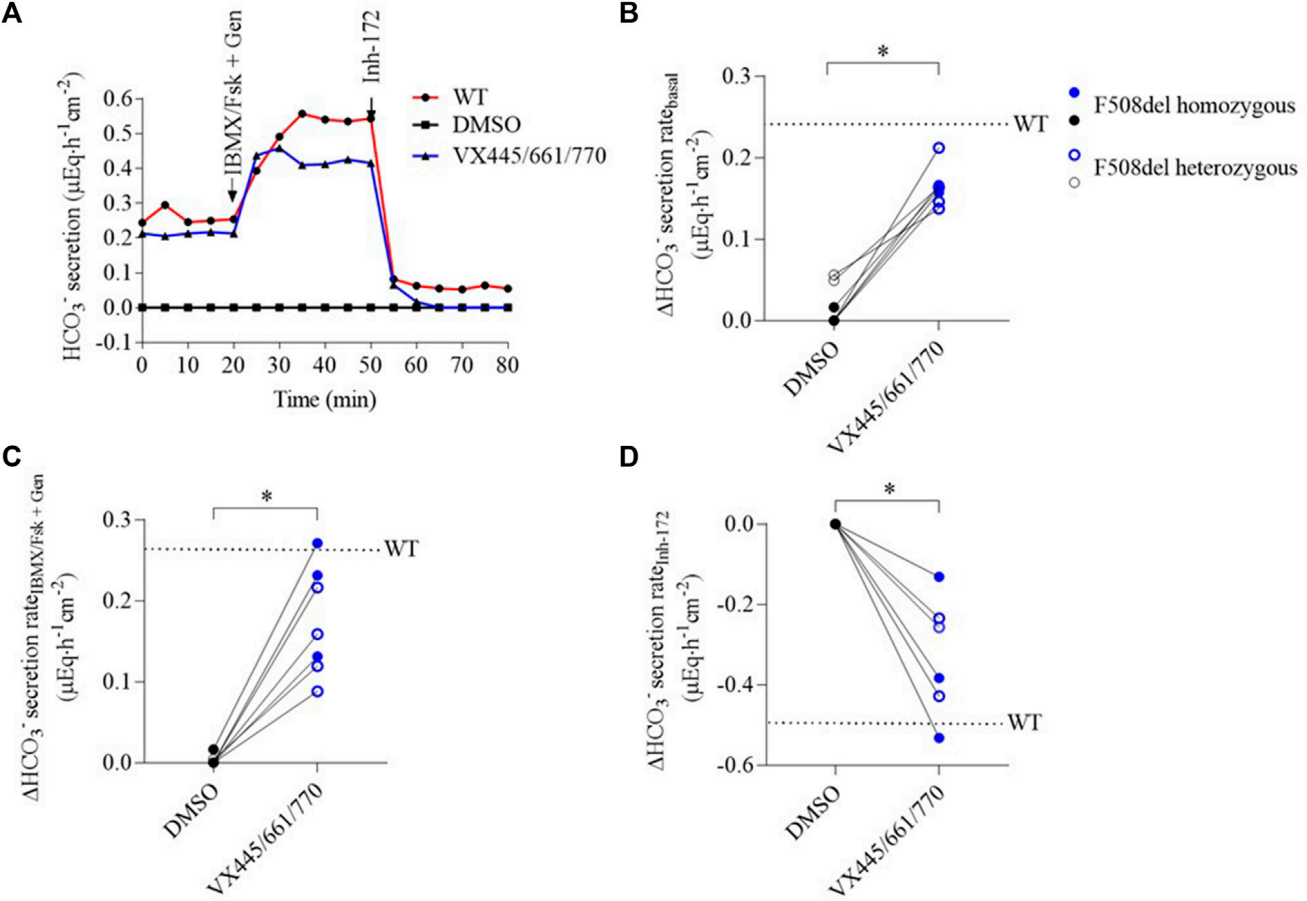
VX-445/661/770 increases F508del-CFTR bicarbonate secretion in respiratory epithelial cells. HCO_3_^−^ flux is measured in WT and F508del airway epithelia by pH-stat at baseline in the presence of amiloride and after the successive addition of Fsk/IBMX (10µM/100 µM) + Gen (100 µM) and Inh-172 (10 µM). Tracings were recorded in WT cells (red) and CF cells after a 48-h incubation with DMSO (black) or VX-445 (3 µM)/VX-661 (3 µM)/VX-770 (100 nM) (blue). **(A)** Representative tracing of the pH-stat experiment of human respiratory epithelial cells from healthy control and a patient carrying a homozygous F508del mutation. **(B)** Individual HCO_3_^−^ secretion rate changes in the presence of amiloride (100 µM) between DMSO- and VX-445/661/770-treated human respiratory epithelial cells (n = 8). The dotted line represents the mean baseline secretion rate of WT cells. F508del homozygous human respiratory epithelial cells are represented as full circles and heterozygous cells as open circles. **(C)** Individual HCO_3_^−^ secretion rate changes in response to Fsk/IBMX + genistein after a 48-h incubation of human respiratory epithelial cells (n = 7) with DMSO or VX-445/661/770. The dotted line represents the mean of WT secretion rate change. **(D)** Individual HCO_3_^−^ secretion rate changes in response to Inh-172 after a 48-h incubation of human respiratory epithelial cells (n = 7) with DMSO or VX-445/661/770. The dotted line represents the mean of the WT secretion rate change. A comparison was carried out by the Wilcoxon matched-pair signed-rank test.*p < 0.05.

Representative tracings of HCO_3_^−^ fluxes in F508del human respiratory epithelial cells under DMSO and VX-445/661/770 conditions are shown in Figure 2A. Control DMSO-treated airway epithelia displayed no HCO_3_^−^ secretion either in the presence of amiloride or after CFTR activation by Fsk/IBMX + genistein. In contrast, VX-445/661/770 treatment elevated HCO_3_^−^ secretion, reaching 78% ± 12% (0.18 ± 0.03 µEq∙h^-1^∙cm^-2^) of the secretion level observed in WT human respiratory epithelial cells (Figures 2A, B). The addition of Fsk/IBMX + genistein increased CFTR-dependent HCO_3_^−^ secretion up to 70% ± 29% of the WT level (Figures 2A, C, and Supplementary Table S5). Inh-172 completely inhibited this HCO_3_^−^ secretion, providing evidence that it was due to CFTR (Figures 2A, D, and Supplementary Table S5). There was no significant difference in HCO_3_^−^ secretion rates between homozygous and heterozygous F508del cells (Figures 2B, C, D, and Supplementary Table S4).

### 3.2 Inflammation enhances CFTR HCO_3_^−^ transport correction in F508del human respiratory epithelial cells

**FIGURE 3:**
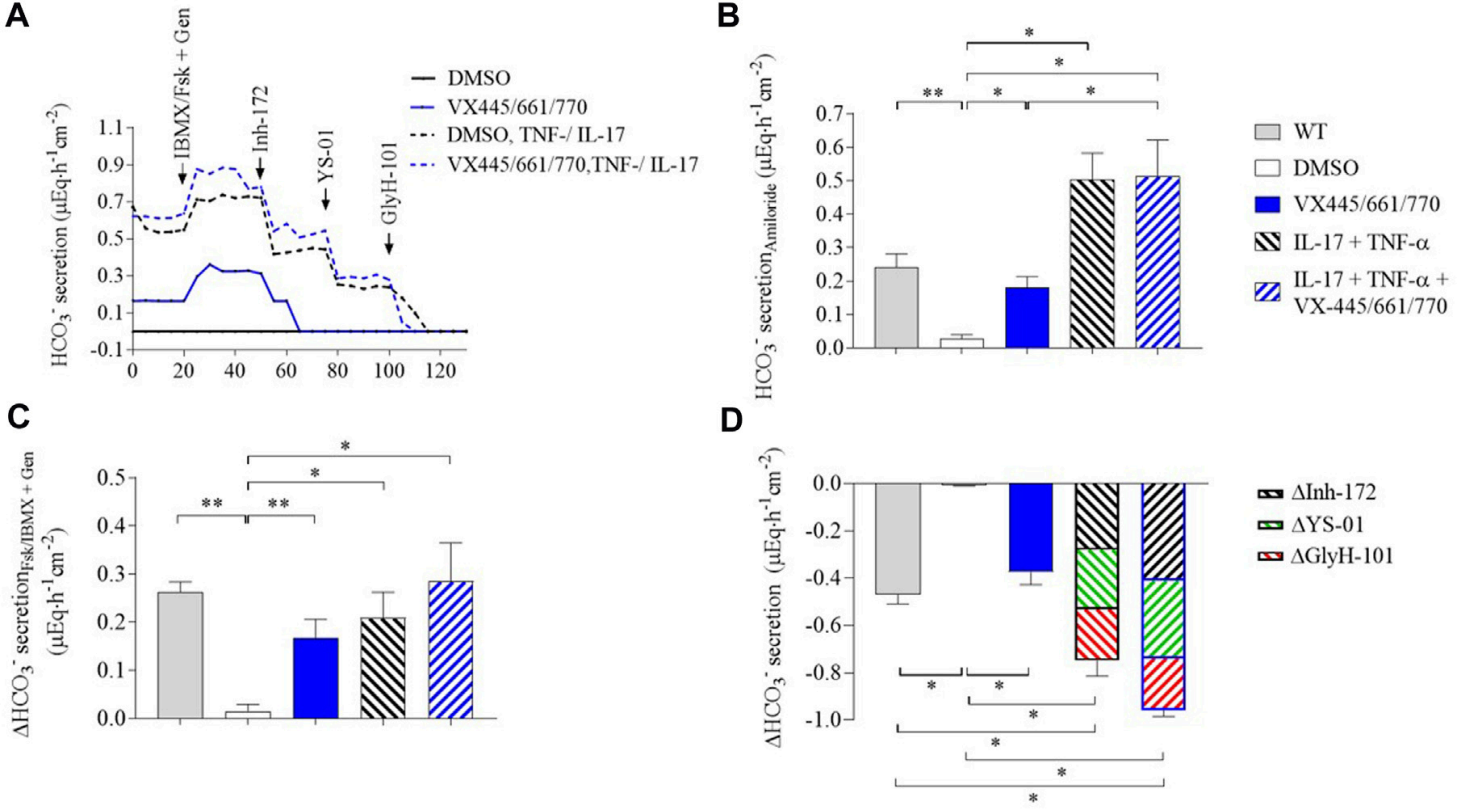
Inflammation increases bicarbonate transport in human respiratory epithelial cells. HCO_3_^−^ flux is measured in WT and F508del human respiratory epithelial cells by pH-stat at baseline in the presence of amiloride and after the successive addition of Fsk//IBMX (10µM/100 µM) + genistein (100 µM) and Inh-172 (10 µM), YS-01 (5 µM), and GlyH-101 (10 µM). CF epithelia were from two F508del homozygous patients and two F508del patients in *trans* with a minimal function mutation (R560K and 1717 + 1G>T). Tracings were recorded after 48-h incubation with DMSO (black line), VX-445 (3 µM)/VX-661 (3 µM)/VX-770 (100 nM) (blue plain line), TNF-α(10 ng/mL)/IL-17 (20 ng/mL) (black dashed line), and TNF-α/IL-17 + VX-445/661/770 (blue dashed line). Representative tracing of the pH-stat experiment of human respiratory epithelial cells from an F508del homozygous patient. HCO_3_^−^ secretion in the presence of amiloride in WT and CF human respiratory epithelial cells after a 48-h incubation with DMSO, VX-445/661/770, TNF-α/IL-17, and TNF-α/IL-17 + VX-445/661/770. HCO_3_^−^ secretion rate changes in response to Fsk/IBMX + genistein in WT and CF human respiratory epithelial cells after a 48-h incubation in DMSO, VX-445/661/770, TNF-α/IL-17, and TNF-α/IL-17 + VX-445/661/770. **(D)** HCO_3_^−^ secretion rate changes after Fsk/IBMX + genistein activation in response to Inh-172 (black lines), YS-01 (green lines), and GlyH-101 (red lines) in WT and CF human respiratory epithelial cells after a 48-h incubation in DMSO, VX-445/661/770, TNF-α/IL-17, and TNF-α/IL-17 + VX-445/661/770. A comparison was carried out by the Mann–Whitney signed-rank test for WT *versus* CF cells and the Wilcoxon matched-pair signed-rank test for comparisons between CF cells. *p*-values are as follows: *p < 0.05; **p < 0.01; other comparisons were not significant.

We next investigated the effect of inflammation alone and in combination with VX-445/661/770 on HCO_3_^−^ secretion (Figure 3, Supplementary Table S5). Incubation with IL-17/TNF-α increased HCO_3_^−^ secretion above the WT level in the presence of amiloride (comparison with WT and NS) (Figures 3A, B, Supplementary Table S5). The change after stimulation by Fsk/IBMX + genistein was up to 87% ± 41% of the WT (Figures 3A, C, Supplementary Table S5). HCO_3_^−^ flux was partially inhibited by Inh-172, and complete inhibition was achieved by the subsequent addition of YS-01 (SLC26A4 inhibitor) and GlyH-101 (respective part in the inhibition of Inh-172, YS-01, and GlyH-101: 37% ± 2%; 33% ± 1.5%; and 29% ± 1%) (Figures 3A, D, and Supplementary Table S5).

The combination of VX-445/661/770 with TNF-α and IL-17 did not modify the amiloride HCO_3_^−^ secretion rate or the cAMP-activated HCO_3_^−^ flux, as compared to TNF-α/IL-17 alone. In contrast, HCO_3_^−^ flux inhibition was increased, mainly the Inh-172 response (*p* < 0.05) (respective part in the inhibition of Inh-172, YS-01, and GlyH-101: 45% ± 2%; 29% ± 6%; and 26% ± 1.5%).

### 3.3 Inflammation, but not VX-445/661/770 alone, induces ASL pH alkalinization

**FIGURE 4:**
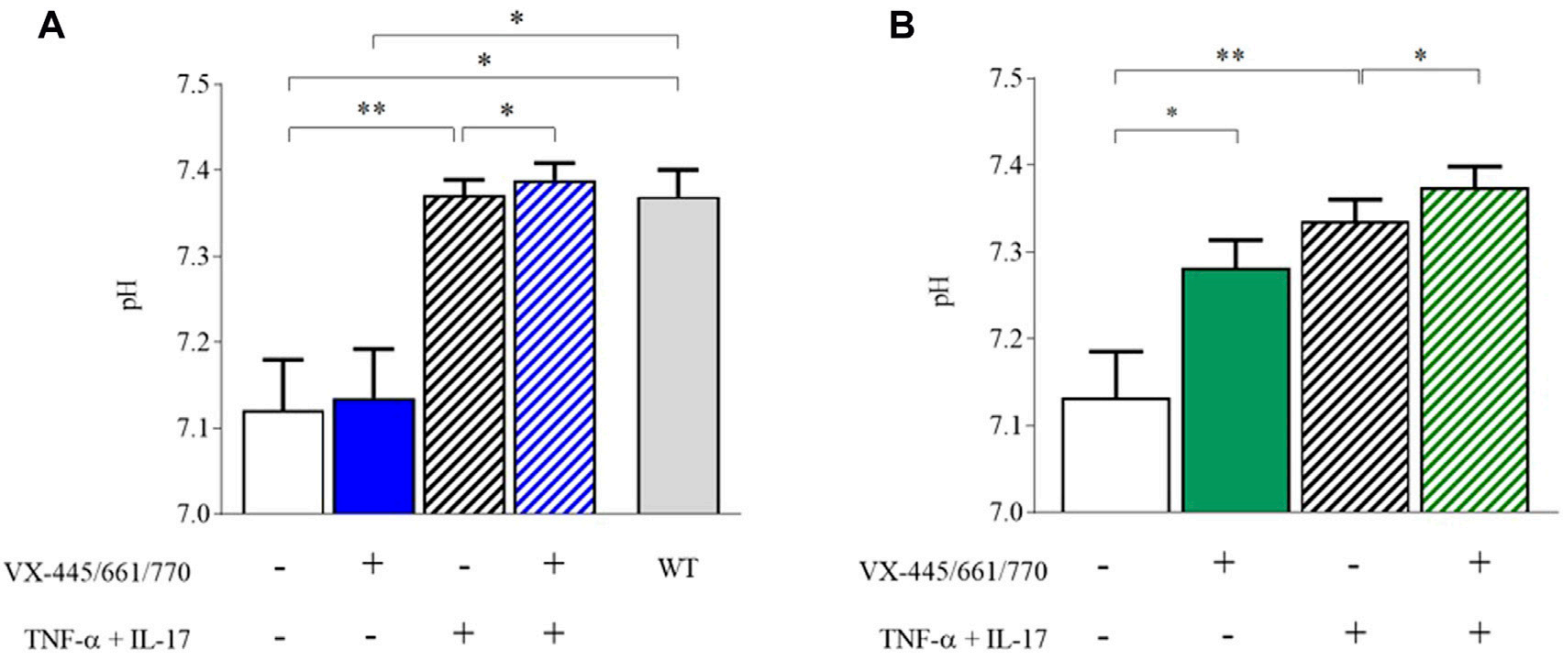
ASL pH of F508del human respiratory epithelial cells is abnormally acidic and is increased by the VX-445/661/770 and TNF-α+IL-17 combination. ASL pH of human respiratory epithelial cells was measured under physiological conditions (37°C, 5% CO_2_) after an 8-h apical incubation of 50 µL physiological Ringer’s solution **(A)** or Cl^−^-free Ringer’s solution **(B)**. The measurements were performed in the presence of Fsk//IBMX (10µM/100 µM) added basolaterally before the 8-h incubation. ASL pH was assessed under physiological conditions in human respiratory epithelial cells from F508del homozygous patients under control DMSO conditions (n = 4) *versus* VX-445/661/770 (n = 4), IL-17+TNF-α (n = 6), IL-17+TNF-α + VX-445/661/770 (n = 6), and WT (n = 5). **(B)** ASL pH was assessed in Cl^−^-free conditions in human respiratory epithelial cells from F508del homozygous patients under control DMSO conditions (n = 9) *versus* VX-445/661/770 (n = 15), IL-17+TNF-α (n = 9), and IL-17+TNF-α + VX-445/661/770 (n = 8). Data are presented as mean ± SD. A comparison was carried out by the Mann–Whitney signed-rank test for WT *versus* CF cells and the Wilcoxon matched-pair signed-rank test. *p*-values are as follows: *p < 0.05; **p < 0.01.

VX-445/661/770 did not significantly change ASL pH in comparison to DMSO control conditions (from 7.12 (0.11) to 7.14 (0.11)) (Figure 4A). In contrast, TNF-α and IL-17 significantly increased ASL pH to 7.37 (0.04) (*p* = 0.009), as shown in Figure 4A. The combination of VX-445/661/770 with TNF-α/IL-17 induced a further small but significant change in ASL pH to 7.39 (0.01) (*p* = 0.03), similar to values observed in WT cells (Figure 4A).

To investigate the respective roles of CFTR and SLC26A4 in pH homeostasis, we performed ASL pH measurements in Cl^−^-free solutions where SLC26A4 activity was abrogated. Under these conditions, the ASL pH of DMSO-treated F508del airway cells was not significantly modified (7.12 vs. 7.14 in Cl^−^ conditions), while it significantly increased from 7.14 (0.11) to 7.28 (0.12) when the cells were incubated with VX-445/661/770, thus unmasking a CFTR-dependent pH correction (Figure 4B). Under pro-inflammatory conditions, ASL pH was significantly increased up to 7.34 (0.08) (*p* = 0.0023 *versus* DMSO but NS *versus* VX-445/661/770), with an additional effect of VX-445/661/770 reaching values similar to those obtained under physiological conditions (7.38 (0.07)).

### 3.4 Inflammation increases CFTR and SLC26A4 expression

**FIGURE 5:**
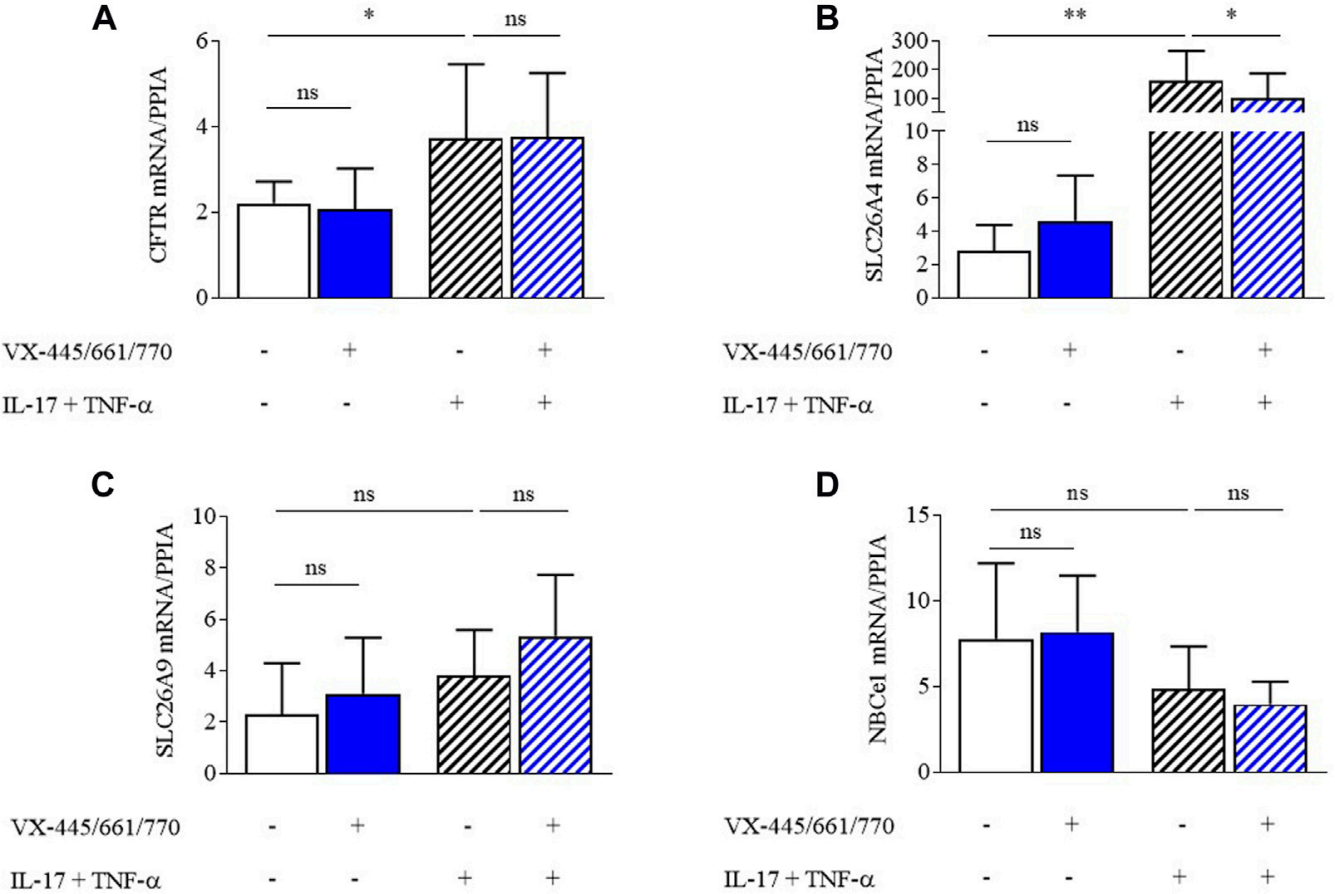
Main base transporter expression in primary F508del/F508del human respiratory epithelial cells by RT-qPCR. Transcript expression levels of *CFTR* **(A)**, *SLC26A4* (Pendrin) **(B)**, *SLC26A9* **(C)**, and *NBCe1* **(D)** were analyzed using real-time RT–PCR in ALI-differentiated primary human bronchial epithelial cells from F508del homozygous patients. Transcript levels were analyzed in the control DMSO condition (n = 8, full white bars), VX-445/661/770 (n = 4, full blue bars), IL-17+TNF-α (n = 8, stripped white bars), and IL-17+TNF-α + VX-445/661/770 (n = 8, stripped blue bars) and normalized with cyclophilin transcript (*PPIA*) as an internal control. When indicated, cells were treated with IL-17 (20 ng/mL) and TNF-α (10 ng/mL) and/or VX-770 (100 nM), VX-445 (3 µM), and VX-661 (3 µM) for 48 h. Data are presented as mean ± SD, and statistical analysis is performed with the Wilcoxon matched-pair signed-rank test (*p < 0.05, **p < 0.01; ns: non-significant).

VX-445/661/770 did not modify the transcript levels of *CFTR, SLC26A4*, *SLC26A9*, or *NBCe1* (Figure 5). TNF-α/IL-17 incubation increased *SLC26A4* transcript levels by more than 40-fold (*p* < 0.001), but in contrast, *CFTR* transcript levels only increased by 1.5-fold (*p* < 0.01), while it did not significantly change *NBCe1* transcript levels. For *SLC26A9,* there was a trend for an increase in the transcript levels upon TNF-α/IL-17; however, this did not reach a significant level (Figure 5).

## 4 Discussion

There are still few pharmacological data on the rescue of HCO_3_^−^ secretion by CFTR correction in the CF airway epithelium. Our results show that F508del-CFTR correction by VX-445/661/770 rescues HCO_3_^−^ transport up to 80% of the normal level in primary cultures of the airway epithelium. Inflammation, e.g., the TNFα/IL-17 cocktail, massively increases the HCO_3_^−^ transport, involving both CFTR and SLC26A4. CFTR correction in the context of inflammation further increased HCO_3_^−^ secretion by CFTR. Strikingly, the increase in CFTR-mediated HCO_3_^−^ secretion had a marginal impact on ASL pH in contrast to TNF-α/IL-17-induced inflammation.

### 4.1 F508del-CFTR correction restores HCO_3_^−^ transport but not ASL pH

Similar observations were recently reported in intestinal F508del organoids where VX-445/661/770 induced an increase in CFTR-dependent HCO_3_^−^ transport (Bijvelds et al., 2022; Ciciriello et al., 2022) and in D1152H primary respiratory cells where ivacaftor improved selective HCO_3_^−^ transport defects (Laselva et al., 2020). Interestingly, in these reports, as shown in our study, carrying F508del on both alleles did not increase the level of HCO_3_^−^ transport correction in comparison to carrying F508del in combination with another mutation. All these results were obtained from short-circuit currents in the Cl^−^-free medium, which enables indirectly assessing HCO_3_^−^ transport by assessing the transepithelial potential difference. Here, by using the pH-stat titration system, an indirect method for assessing HCO_3_^−^ secretion, we demonstrate that VX-445/661/770 treatment increases net transepithelial HCO_3_^−^ fluxes. The increase in secretion in response to Fsk + genistein and its complete inhibition by Inh-172 indicate that this effect is due to CFTR.

Nevertheless, despite this massive increase in CFTR-dependent HCO_3_^−^ transepithelial transport, the effect of F508del-CFTR rescue on the ASL pH of cultured airway epithelia was marginal. This confirms the observations by Rehman et al. (2021) and Morrison et al. (2022) and provides evidence that the correction of the CFTR channel defect alone is not sufficient to buffer the acidic ASL of CF airways. In contrast, Ludovico et al. showed that VX445/661/770 alkalinized the pH to WT levels (Ludovico et al., 2022). The reason for this discrepancy is unclear but may be related to differences in the media culture and differential expression of HCO_3_^−^ transporters (Saint-Criq et al., 2020; Livnat et al., 2023).

### 4.2 Functional interplay between CFTR and SLC26A4

Airway inflammation is a hallmark of CF disease observed from the first days of life, mainly driven by neutrophilic inflammation and various cytokines such as TNF-α and IL-17 (Lukacs et al., 1995; Tan et al., 2011; Sly et al., 2013). The TNFα/IL-17 inflammatory cytokine combination not only modulates innate anti-infective defense (McAleer and Kolls, 2011) but also our data point to a massive increase in HCO_3_^−^ secretion both at the basal state and after CFTR activation by cAMP (Fsk/IBMX). In addition, pH-stat experiments enabled us to dissect the participation of other transporters in ASL pH homeostasis. They clearly highlighted the SLC26A4 electroneutral Cl^−^/HCO_3_^−^ exchange, as the complete inhibition of HCO_3_^−^ flux required the subsequent addition of the YS-01 SLC26A4 inhibitor (Lee et al., 2020) and GlyH-101. This pro-inflammatory condition was associated with the pH normalization of F508del ASL. Our results are consistent with Rehman et al.’s observations (Rehman et al., 2020) indicating that SLC26A4 is a key player in CF ASL alkalinization and the recent publication of Guidone et al. showing that YS-01 significantly decreased ASL pH in both CF and non-CF epithelia treated with IL-17/TNF-α (Guidone et al., 2022). However, in contrast to our studies, Guidone et al. observed significant ASL pH alkalinization only after the stimulation of IL-17/TNF-α-treated cells by isoproterenol (Guidone et al., 2022). This discrepancy might be related to cell culture media that impact gene expression and the activity of ion channels (Saint-Criq et al., 2020). As TNF-α/IL-17 increases SLC26A4 and CFTR transcript expression, one could hypothesize that the modification of pH, driven by inflammation, could be related to SLC26A4 activity, both directly by increasing HCO_3_^−^ secretion by SLC26A4 and CFTR and indirectly via its functional coupling to corrected F508del-CFTR, which would fuel SLC26A4 with Cl^−^ (Garnett et al., 2011; Kim et al., 2019). SLC26A4 appears, therefore, as a key player in pH homeostasis under inflammatory physiological conditions. However, the observation that the correction of CFTR by modulators further increases HCO_3_^−^ secretion by CFTR under inflammatory conditions and that pH is significantly increased in the Cl^−^-free medium where SLC26A4 is defective unravels the role of CFTR in ASL pH homeostasis.

The fact that GlyH-101 was necessary to completely inhibit the HCO_3_^−^ transport in the context of inflammation suggests that additional HCO_3_^−^ transporters other than CFTR and SLC26A4 may be involved in this inflammation-induced alkalinization (Zajac et al., 2021; Liu et al., 2022). Indeed, while Inh-172 is reported as being very specific to CFTR gating (Caci et al., 2008), GlyH-101 not only occludes the CFTR pore but is also active on anoctamin, bestrophin (Bai et al., 2021), voltage-gated channels (Lin et al., 2023), and SLC26A9 (Bertrand et al., 2009; Liu et al., 2022). It is still debated whether SLC26A9 contributes to HCO_3_^−^ secretion directly, as suggested by a recent study on perfused mouse bronchioles and nasal cells (Jo et al., 2022; Liu et al., 2022), or via reciprocal regulation with CFTR, as suggested by the observation that the correction of F508del-CFTR also restored SLC26A9 constitutive activity in airway cells (Bertrand et al., 2009; Bertrand et al., 2017; Larsen et al., 2021). Our experiments show that SLC26A9 transcripts are present in the airways and display a trend to increase upon inflammatory triggers, but not at a significant level. This increase may be responsible for the stabilization of corrected F508del-CFTR at the plasma membrane, as shown by Pinto et al. (2021), and thus contribute indirectly to the higher HCO_3_^−^ fluxes mediated by CFTR observed in pH-stat experiments (Balázs and Mall, 2018).

Paracellular HCO_3_^−^ flux might also control ASL pH. Very well-designed studies by Thornell et al. (2020) have shown that in human airway epithelia, there is a paracellular pathway permeable to HCO_3_^−^ ions that may temper the ASL pH changes. Depending on the values of ASL pH, the paracellular flux is absorptive (ASL pH 7.4) or secretory (ASL pH 6.6). Interestingly, cytokine treatment (IL-17/TNF-α) resulted in an increase in paracellular conductance and HCO_3_^−^ permeability, suggesting that the modulation of paracellular HCO_3_^−^ transport might represent new therapeutic targets (Parker, 2020). In our pH-stat experiments, we did not observe a paracellular HCO_3_^−^ flux, as we were able to completely block HCO_3_^−^ transport by using ion-transporting protein inhibitors. This is consistent with Thornell’s observations (Thornell et al., 2020) showing negligible paracellular HCO_3_^−^ flux at pH 7.0 (at which we clamp our apical solution).

### 4.3 Physiopathological relevance of our findings

Altogether, our results suggest that, under physiological conditions, SLC26A4 is the main driver of pH regulation in the bronchial epithelium, with CFTR intervening indirectly by fueling the exchanger with Cl^−^. Nevertheless, CFTR HCO_3_^−^ secretion may be required in specific acidic situations, such as gastric acid inhalation, to efficiently and rapidly buffer ASL pH. This has been shown in murine duodenum, where low luminal pH activates the transcellular HCO_3_^−^ transport that requires CFTR (Hogan et al., 1997). Moreover, the increased levels of HCO_3_^−^ into the airway microenvironment, due to a proximal paracrine effect, would also favor rapid mucin expansion, resulting in improved mucociliary clearance and decreased airway obstruction and inflammation (Quinton, 2008). Downstream effects could also restore intrinsic immune defenses by augmenting the formation of neutrophil extracellular traps by neutrophils and sensitizing *Pseudomonas aeruginosa* to the antimicrobial peptide cathelicidin LL-37 (Siew et al., 2022).

These results suggest that the full efficiency of CFTR modulation might require a residual “background” of CFTR inflammation. This is consistent with *in vivo* studies that showed that nasal mucosa pH was abnormally acidic in CF neonates but normal from 3 months of age after airway inflammation initiation (Abou Alaiwa et al., 2014; Schultz et al., 2017), and a positive correlation between the level of airway inflammation and the respiratory improvement in G551D patients treated with ivacaftor was reported by Rehman et al. (2021). Importantly, several studies report that CFTR modulators abrogate airway inflammation, which may then jeopardize the effect on pH (Lepissier et al., 2023). The physiopathological relevance of these findings, including their long-term remanence, is still unclear (Hisert et al., 2017).

### 4.4 Study limitation: current knowledge and technical gaps

This study has limitations. First, we measured ASL pH with a pH electrode and assumed that the 50-µL Ringer solution added to the surface of airway cultures for the experiment would represent diluted ASL. However, this ringer contains 25 mM HCO_3_^−^ and could buffer small pH variations and, thus, underestimate pH changes.

Second, inflammation is a complex status involving different triggers that may behave differently (Roesch et al., 2018; Gentzsch et al., 2021; Rehman and Welsh, 2023). Moreover, the concentration of cytokines used in this experiment might be far above that occurring *in vivo*. Indeed, we performed a pilot study to assess inflammatory cytokines in sputum and observed that TNF-α levels were 100-fold lower in patients’ sputum than the concentration applied *in vitro* (Lepissier et al., 2023). Our experimental design is only able to mimic acute inflammation. It could be noted that CFTR rescue may differentially modulate CF epithelia subjected to chronic inflammation. Additionally, we used the novel YS-01 inhibitor as it was shown to potently inhibit pendrin activity with no effects on other ion-transporting proteins involved in ASL regulation such as CFTR or ANO-1; however, its possible off-target effects are still to be discovered (Park et al., 2019; Lee et al., 2020).

Third, we measured the HCO_3_^−^ transport under conditions aiming to maximize HCO_3_^−^ fluxes. To do this, we used the ENaC channel inhibitor (amiloride) to hyperpolarize cell membranes and, thus, increase the driving force for HCO_3_^−^ exit. Solution at the apical side of cell monolayers was unbuffered and therefore generated a large transepithelial (basal to apical) HCO_3_^−^ gradient with a negligible paracellular HCO_3_^−^ flux (Thornell et al., 2020). The apical solution pH was clamped to 7.00 to reflect the pH value found in CF patients (Zajac et al., 2021). Finally, we applied a CO_2_/O_2_ gradient to the epithelium and used a solution with no HCO_3_^−^ to be able to monitor the HCO_3_^−^ fluxes. We agree that the use of an apical pure O_2_ and HCO_3_^−^ gradient is not physiological and might impact airway cell physiology. However, we do not think that the O_2_ level would affect intracellular pH. Indeed, we did not observe any short-term (3–4 h) modification of transepithelial resistance, and Liu et al., under conditions relatively similar to ours, did not report long-term modification of intracellular pH in mouse bronchioles (Liu et al., 2022). Moreover, different groups showed that such an HCO_3_^−^ gradient did not cause an overestimation of HCO_3_^−^ secretion (Krouse et al., 2004; Shan et al., 2012).

Finally, our pH-stat experiments show net HCO_3_^−^ secretion as concurrent H^+^ transport neutralizes part of the transported HCO_3_^−^. Thus, if present, H^+^ transport may lead to an underestimate of our HCO_3_^−^ fluxes. This was clearly highlighted in experiments performed in the Calu-3 cell line, showing that HCO_3_^−^ secretion may be 30% higher than the measured net flux (Krouse et al., 2004; Shan et al., 2012). Our experiments measure global luminal pH and HCO_3_^−^ transport but may underestimate local pH modification in micro areas around submucosal glands, which express CFTR at a high level and display an acidic pH environment related to CFTR defects (Garnett et al., 2016). Local correction of HCO_3_^−^ secretion may be very relevant to acting on inflammation-mediated mucus viscosity. ASL acid–base regulation is a very complex phenomenon involving the coordinated activity of all ion-transporting proteins, including H^+^-transporting proteins present on the apical and basolateral sides of the epithelium (Zajac et al., 2021). The measurement of ASL pH cannot discriminate between the increase in base secretion and the decrease in acid secretion (Shah et al., 2016). The regulation of the ATP12A proton pump by inflammation, as recently reported by Guidone et al., showed that the impact on H^+^ secretion also needs to be considered (Guidone et al., 2022).

### 4.5 Bicarbonate transport correction: a path to theratyping

Despite the revolution in CF therapy caused by HEMT, real-world studies have shown high variability in patient responses to modulator therapy (Pranke et al., 2017; Birimberg-Schwartz et al., 2023; Dreano et al., 2023). Theratyping gives the possibility to examine the responses of CFTR modulator drugs *in vitro*, providing the opportunity to individualize medicine for pwCF (Mall et al., 2020). Our laboratory has implemented theratyping based on HNE cells generated from the patient’s nasal brushes (Bear and Ratjen, 2023; Dreano et al., 2023). Our initial pH-stat studies have shown that the HNE cell monolayers recapitulate the HCO_3_^−^ transport properties of HBE cultures, and thus, this model is useful in showing patient-specific properties and responses to drug therapy. This could be especially interesting since some *CFTR* variants were shown to be mainly HCO_3_^−^ (and not Cl^−^) defective (LaRusch et al., 2014). It may be that patients carrying genotypes associated with residual Cl^−^ secretion improve because of the rescue of HCO_3_^−^ secretion. This aspect has been underestimated until now, and bicarbonate rescue now needs to be put on the spot. Moreover, since inflammation is a hallmark of CF, it is mandatory to test CFTR ion transport and, more specifically, HCO_3_^−^ transport during inflammatory conditions (Gentzsch et al., 2021).

## 5 Conclusion

Our data show that CFTR correction restores HCO_3_^−^ secretion, but at a level that is not sufficient to overcome ASL pH acidosis. Inflammation is a key regulator of HCO_3_^−^ secretion in CF airways and enhances the efficacy of CFTR modulators. The prediction of the response by theratyping should take into account the airway inflammatory phenotype and its effect on ASL pH.

## Data Availability

The raw data supporting the conclusions of this article will be made available by the authors without undue reservation.
